# A Cross-Sectional Study on the Prevalence of Depression and Associated Factors in Tuberculosis Patients in the Vidisha District of Madhya Pradesh, India

**DOI:** 10.7759/cureus.46637

**Published:** 2023-10-07

**Authors:** Naresh Solanki, Ajay Upadhyay, Rajkumar Upadhyay, Deepti Dabar, Uday K Mandal, Vikas Yadav, Sanjay S Agarwal

**Affiliations:** 1 Department of Psychiatry, Atal Bihari Vajpayee Government Medical College, Vidisha, IND; 2 Department of Tuberculosis and Respiratory Medicine, Atal Bihari Vajpayee Government Medical College, Vidisha, IND; 3 Department of General Medicine, People’s College of Medical Sciences & Research Centre, Bhopal, IND; 4 Department of Community and Family Medicine, All India Institute of Medical Sciences, Bhopal, IND; 5 Department of Environmental Health and Epidemiology, Indian Council of Medical Research (ICMR) - National Institute for Research in Environmental Health, Bhopal, IND; 6 Department of Community Medicine, Atal Bihari Vajpayee Government Medical College, Vidisha, IND

**Keywords:** mental health, prevalence, india, tuberculosis, depression

## Abstract

Introduction: Tuberculosis (TB) is a major public health problem in the developing world. Depression affects medicine adherence in TB patients. There is a scarcity of data regarding the prevalence of depression among TB patients from any city in central India. Therefore, the aim of this research was to study the prevalence of depression and associated factors in TB patients in the Vidisha district of Madhya Pradesh, India.

Methods: This was a cross-sectional study conducted on 106 TB patients visiting the TB and chest outpatient department of Atal Bihari Vajpayee Government Medical College (ABVGMC). It is a tertiary health care facility located in the district of Vidisha in Madhya Pradesh, India. Data collection was done from September 2020 to January 2021. Depression was measured using the Patient Health Questionnaire-9 (PHQ-9). We used a semi-structured questionnaire to collect data regarding relevant demographic and behavioral factors. Analyses were done in IBM SPSS software, version 25 (IBM Corp., Armonk, NY).

Results: The prevalence of depression among TB patients was 55.7% (n = 59). Depression in the mild category was most common (n = 44, 41.5%), followed by moderate (n = 10, 9.4%), and the moderately severe (n = 5, 4.7%) category. Depression prevalence was found to be slightly more common in females (58.5% vs. 52.8% in males), married participants (58.2% vs. 51.3% in unmarried), educated more than high school (56.8% vs. 54.8% in less than high school), socioeconomically Above Poverty Line (APL) (60.5% vs. 52.4% in Below Poverty Line (BPL)), living in urban areas (60.9% vs. 47.6% in rural areas), and in the continuation phase of anti-TB treatment (58.6% vs. 52.1% in intensive phase), but differences were statistically non-significant. Depression was significantly associated with the medicine non-adherence group (vs. the medicine adherence group; p-value: 0.022) and the previously treated TB patient category (vs. the new case group; p-value: 0.031).

Conclusion: The prevalence of depression among TB patients was very high (55.7%). The prevalence of depression was significantly higher in the medicine non-adherent group (p-value: 0.022) and the previously treated TB patient group (p-value: 0.031). In this study, we have not found any significant association between the prevalence of depression among TB patients and sex, marital status, education attainment, poverty status, or living in an urban or rural areas.

## Introduction

Tuberculosis (TB) is still a major public health problem in developing countries like India. In 2017, India was responsible for a quarter of the total number of TB cases globally [1]. In 2015 alone, among the estimated total incidence of 2.8 million cases, nearly half a million people died due to TB in India [2].

Depression is characterized by loss of interest or pleasure, sadness, feelings of guilt or low self-worth, poor concentration, and feelings of tiredness [3]. Globally, depression affects almost 350 million people and is one of the most significant contributors to the ‘global burden of disease’ in the young and middle-aged (15-45 years) [4].

Several studies have shown that the prevalence of psychiatric disorders, particularly depression, is high among TB patients [5]. Studies also reported that TB patients are at higher risk of developing psychological problems [6]. Tuberculosis patients who were found to be depressed when treated with cognitive therapy have resulted in a lower percentage of defaulters and an increased number of treatment completions [7]. A TB patient might develop depression due to the long duration of treatment, stigmatization, and lack of family support.

Examining the prevalence of depression and its associated factors among tuberculosis patients holds significance, as addressing depression enhances treatment adherence and, subsequently, treatment completion rates in this population [7]. Furthermore, there is a lack of such data from any district in central India. Hence, it becomes imperative to investigate the prevalence of depression and its associated factors in TB patients in this region. This effort lays the foundation for future research to develop interventions aimed at addressing or preventing these risk-increasing circumstances. This was the primary objective of our current study.

## Materials and methods

This is a cross-sectional study conducted among TB and chest outpatient department (OPD) attendees of Atal Bihari Vajpayee Government Medical College (ABVGMC) in Vidisha, Madhya Pradesh, India. Atal Bihari Vajpayee Government Medical College is a tertiary healthcare facility with approximately 1100 daily outpatients for all ailments, out of which 80 patients attended TB and chest OPD. Attendees of TB and chest OPD who had TB and were aged more than 18 years formed the study population. Inclusion criteria for participation in this research were previously diagnosed patients of TB (any type) as per the guidelines of the Revised National TB Control Programme (RNTCP) (disease duration greater than 15 days) [8]. Participants aged less than 18 years, attending OPD due to other ailments, and not providing consent were excluded from the study.

Screening for depression was done using the Hindi version of the Patient Health Questionnaire (PHQ-9) [9]. It has been validated in the Indian population and is considered a reliable tool for diagnosing depression [10]. The PHQ-9 is used to make a provisional diagnosis of depressive disorder and provides a severity score for depressive disorder. On the basis of the scores obtained in this questionnaire, participants were classified into the following categories: no depression (score 0-4), mild depression (score 5-9), moderate depression (score 10-14), moderately severe depression (score 15-19), and severe depression (score 20-27). In that sense, a patient obtaining a score of five or more is considered to have depression. Each questionnaire took approximately 10-15 minutes to fill out. A PHQ-9 score of 10 or greater was found to have a sensitivity for major depression of 88% and a specificity of 88% [11,12].

Medicine adherence was measured by asking for missing dose information in the last week. Participants who missed any medicine dose were considered non-adherent to treatment [13]. To consider participants’ poverty status, Below Poverty Line (BPL) cards were asked to be shown. The government of India issues BPL cards to households living before the poverty line after an assessment of their poverty status [14]. Urban or rural residential status was determined by categorizing their addresses using the definitions of the 2011 India Census [15].

According to RNTCP guidelines, TB treatment is provided to patients in two phases: the initiation phase and the continuation phase. New cases are those TB patients who were never treated for TB or treated for less than 28 days. Whereas previously treated TB patients are those who have taken more than 28 days of anti-TB treatment previously [8].

All consecutive patients with TB were referred to the psychiatry OPD for assessment. Considering 35% (Kumar K et al.) prevalence and 10% allowable error, the sample size was calculated to be 85 (with a formula 1.952PQ/L2). Considering 25% rejection, the final sample size was 111 [16].

Previously diagnosed (diagnosed >15 days ago) patients with TB attending TB and respiratory diseases OPD at ABVGMC, Vidisha, were interviewed. We used a semi-structured questionnaire to collect data regarding screening for depression and relevant demographic and behavioral factors.

All data were entered using Google Forms (Google LLC, Mountainview, CA) and exported as a Microsoft Excel sheet (Microsoft Corp., Redmond, WA). Later, the data were imported to IBM SPSS software version 25 (IBM Corp., Armonk, NY) and analyzed. This study’s variables, measured on the Likert scale, were divided into categories using standard cut-offs. Categorical variables were presented as frequency tables, and numerical variables were presented as mean and standard deviation. The association of two categorical variables was assessed using the chi-square test. Statistical significance was considered when the p-value was less than 0.05.

This study was conducted after getting approval from the institutional ethical committee of ABVGMC, Vidisha, Madhya Pradesh, India (Ref. No. 19D/IEC/ABVGMC/Vidisha/2020). All participants were given a participant information sheet in Hindi and were asked to fill out informed written consent in Hindi. All participants with depression were further investigated in the psychiatry OPD for clinical diagnosis and treatment.

## Results

Data collection for this study was conducted from September 2020 to January 2021. We contacted a total of 111 TB patients and were able to collect data from 106 consenting participants. The mean age of the participants was 34.3 years (SD: 13.0 years, range: 18-81 years).

In our study, male and female (n = 53 each, 50% each) participants participated equally. Most of the participants were married (n = 67, 63.2%). Most participants had education up to the tenth grade of schooling (n = 62, 58%). Almost half of the participants were working or doing some job (n = 51, 48.1%), whereas others were homemakers (n = 31, 29.2%) or students (n = 24, 22.6%). In our study, most of the participants socioeconomically belonged to the BPL (n = 63, 59.4%) group, and 40.6% (n = 43) belonged to the Above Poverty Line (APL) group. Many were living in urban areas (n = 64, 60.4%), and 39.6% (n = 42) were living in rural areas (Table 1).

**Table 1:** The demographic characteristics of the study participants

Demographic variable	Characteristics	Number (percentage)
Sex	Male	53 (50)
	Female	53 (50)
Marriage	Married	67 (63.2)
	Unmarried	38 (35.8)
	Widow/Widower	1 (0.9)
Education	Illiterate	18 (17)
	Just literate	21 (19.8)
	Grade 1 to grade 5	7 (6.6)
	Grade 6 to grade 8	7 (6.6)
	Grade 9 to grade 10	9 (8.5)
	Grade 11 to grade 12	24 (22.6)
	Graduate and above	20 (18.9)
Work profile	Working	51 (48.1)
	Homemaker	31 (29.2)
	Student	24 (22.6)
Poverty	Below Poverty Line	63 (59.4)
	Above Poverty Line	43 (40.6)
Living area	Urban	64 (60.4)
	Rural	42 (39.6)

On the assessment of depression with the PHQ-9 scale, most had mild to moderately severe depression (n = 59, 55.7%). Depression in the mild category was most common (n = 44, 41.5%), followed by moderate (n = 10, 9.4%), and moderately severe (n = 5, 4.7%). Most participants adhered to their medicines (n = 81, 76.4%), and only a quarter (n = 25, 23.6%) did not take their medicines regularly. In this study, almost half of the participants were treated as new cases (n = 57, 53.8%) of TB, and 49 (46.2%) participants were treated as previously treated patients of TB. In this research, 48 (45.3%) participants were in the intensive phase, and 58 (54.7%) took the continuation phase of TB treatment (Table 2).

**Table 2:** Disease characteristics of the participants TB: tuberculosis

Disease characteristics	Feature	Number (percentages)
Depression	None	47 (44.3)
	Mild	44 (41.5)
	Moderate	10 (9.4)
	Moderately severe	5 (4.7)
Medicine adherence in the last one week	Yes	81 (76.4)
	No	25 (23.6)
Tuberculosis type	New case	57 (53.8)
	Previously treated patients	49 (46.2)
TB treatment phase	Intensive phase	48 (45.3)
	Continuation phase	58 (54.7)

In this study, we found that depression was slightly more prevalent in female TB patients (n = 31, 58.5%) than in males (n = 28, 52.8%), but the difference was non-significant. More married participants (n = 39, 58.2%) had depression compared to currently single participants (n = 20, 51.3%), though the difference is again statistically non-significant. An almost equal number of participants who were educated up to high school (n = 34, 54.8%) and more than high school (n = 25, 56.8%) were having depression (difference was non-significant). We found that depression levels were higher in APL participants (n= 26, 60.5%) as compared to BPL participants (n = 33, 52.4%), but the difference is statistically non-significant. Though the prevalence of depression is higher in participants in urban areas (n = 39, 60.9%) compared to rural areas (n = 20, 47.6%), the difference was non-significant. In our study, we did not find that confounding factors such as sex, marital status, education level, poverty status, or urban/rural residence were predisposing factors for the prevalence of depression in tuberculosis patients (Table 3).

**Table 3:** Association of various factors with depression * Difference is statistically significant (p-value<0.05). Statistical significance was considered when the p-value was less than 0.05.

Variables	Characteristics (total numbers)	Depression present as no. (%)	Depression absent as no. (%)	Pearson's Chi-square value	p-value (Chi-square test)
Sex	Male (53)	28 (52.8)	25 (47.2)	0.344	0.696
	Female (53)	31 (58.5)	22 (41.5)		
Marriage	Married (67)	39 (58.2)	28 (41.8)	0.479	0.546
	Unmarried/ widow/ widower (39)	20 (51.3)	19 (48.7)		
Education	Up to high school (62)	34 (54.8)	28 (45.2)	0.041	1
	More than high school (44)	25 (56.8)	19 (43.2)		
Poverty	Below Poverty Line (63)	33 (52.4)	30 (47.6)	0.667	0.43
	Above Poverty Line (43)	26 (60.5)	17 (39.5)		
Living area	Urban (64)	39 (60.9)	25 (39.1)	1.823	0.231
	Rural (42)	20 (47.6)	22 (52.4)		
Medicine adherence in the last one week	Non-adherent (25)	19 (76.0)	6 (24.0)	5.48	0.022*
	Adherent (81)	40 (49.4)	41 (50.6)		
Tuberculosis type	New case (57)	26 (45.6)	31 (54.4)	5.043	0.031*
	Previously treated patients (49)	33 (67.3)	16 (32.7)		
TB treatment phase	Intensive phase (48)	25 (52.1)	23 (47.9)	0.455	0.558
	Continuation phase (58)	34 (58.6)	24 (41.4)		

Depression prevalence was higher in the group showing medicine non-adherence (n = 19, 76.0%) as compared to the medicine-adherent group (n = 40, 49.4%), and this difference was statistically significant (p-value: 0.022). Tuberculosis patients in the new case category (n = 26, 45.6%) had less depression as compared to the previously treated patients category (n = 33, 67.3%), and this difference was also statistically significant (p-value: 0.031). Although participants in the continuation phase of the TB treatment group (n = 34, 58.6%) had a slightly higher prevalence of depression than participants in the intensive phase (n = 25, 52.1%) of the TB treatment group, this difference was statistically non-significant (Table 3).

## Discussion

The etiology of the TB and depression association is complex and multifactorial. Psychological and biological mechanisms were suggested for such an association. Studies indicated that TB patients might develop depression due to the mental burden of chronic infection and psycho-socio-economic stressors [17,18]. Anti-TB treatment with isoniazid might also be responsible for the development of depression [19]. Patients with depression may develop TB because of compromised immunity due to neglected self-care [20]. Studies have also suggested that shared risk factors may trigger the concurrent development of TB and depression [21].

In the current research, we collected data from 106 TB patients. The mean age of participants was 34.3 (SD = 13.0) years. Male and female participation was equal (n = 53 and 50% each). The majority of them were married (n = 63%). More than half (n = 62, 58.4%) of them were educated up to the tenth grade of schooling. Most (n = 51, 48.1%) of the participants were doing some job, whereas 29.2% (n = 31) and 22.6% (n = 24) were homemakers and students, respectively. Most (n = 63, 59.4%) of the participants were socioeconomically BPL. Most participants lived in urban areas (n = 64, 60.4%). Most participants had mild to moderately severe depression (n = 59, 55.7%).

The high prevalence of depression found in current research is in agreement with previous research studies conducted worldwide. Duko B. et al. conducted a cross-sectional survey of TB patients and found a very high prevalence of depression among TB patients [17]. Various other studies conducted globally also found a very high prevalence of depression among TB patients [5]. Indian studies were also found to have higher rates of depression in TB patients [16,22].

In this study, we found that depression is more prevalent in female TB patients (n = 31, 58.5%), but the difference was non-significant. In previous research, a significantly higher prevalence of depression in females than in males has been observed in TB patients [22]. In the current study, the power of the study might not be sufficient to conclude decisively.

Worldwide, depression is 1.7 times more prevalent in females than males (general population) [23]. Hormonal changes in women’s lifecycles might be responsible for such a high prevalence [24]. Genetic and social mechanisms have also been suggested for such associations [24].

More married participants (n = 39, 58.2%) had depression compared to currently single participants (n = 20, 51.3%), though the difference is again statistically non-significant. Dahiya S. et al. found that being unmarried is a risk factor for depression [25]. We might have found definitive results in the current study with a larger sample size.

In our study, we did not find any statistically significant difference in the prevalence of depression due to education level (up to high school vs. more than high school), poverty status (below poverty line vs. above poverty line), living area (urban vs. rural), and phase of the TB treatment (initiation phase vs. continuation phase).

Medicine non-adherence was significantly higher in patients with depression (p-value: 0.022). Depression is a known risk factor for medicine non-adherence in various diseases [26]. Depression increases non-adherence due to several mechanisms. Firstly, a certain level of positive expectation and belief in treatment is required for patients’ adherence to medicine, but depression adds considerable hopelessness. Secondly, successful continuation of treatment of chronic diseases requires emotional support from family and friends, but social isolation created by depression may disrupt such mechanisms. Thirdly, a depressed person’s cognition might deteriorate to such an extent that the patient may not be able to remember or follow treatment recommendations [27]. Further research is required to understand non-adherence in patients with depression and to develop approaches to improve adherence.

Patients who have taken anti-TB treatment previously (previously treated patients) are significantly (p-value: 0.031) more associated with depression as compared to first-time (new case) TB patients. Similar observations were documented by Kehbila J. et al. [28]. It might be possible that previously treated patients may have stopped anti-TB treatment at their previous treatment attempt due to depression, a known factor for medicine non-adherence [28].

This cross-sectional study conducted to determine the prevalence of depression in TB patients faces a few limitations. Firstly, the small sample size may not accurately represent the entire population of tuberculosis patients, potentially leading to biased results and reduced generalizability. Moreover, the study's cross-sectional design only provides a snapshot of the participants' mental health at a single point in time, making it difficult to establish causality or determine the temporal relationship between tuberculosis and depression. Finally, relying on self-reported measures to assess depression may introduce response bias and subjective interpretations of participants' mental states. Overall, while the study provides valuable insights, these limitations underscore the need for more comprehensive research to better understand the relationship between tuberculosis and depression.

## Conclusions

We found a very high (n = 59, 55.7%) prevalence of depression among TB patients. Other noteworthy findings were that the prevalence of depression was significantly higher in the medicine non-adherent group (p-value: 0.022) and the previously treated TB patient category (p-value: 0.031). In this study, we did not find any significant association between the prevalence of depression among TB patients and sex, marital status, education attainment, poverty status, or living in an urban or rural area.

Our research results highlighted the need for mandatory screening of depression among TB patients, especially the previously treated patients group, to improve medicine adherence and treatment outcomes.
